# Bacteriocin production enhancing mechanism of *Lactiplantibacillus paraplantarum* RX-8 response to *Wickerhamomyces anomalus* Y-5 by transcriptomic and proteomic analyses

**DOI:** 10.3389/fmicb.2023.1111516

**Published:** 2023-02-24

**Authors:** Rong Nie, Zekang Zhu, Yanwei Qi, Zhao Wang, Haoxuan Sun, Guorong Liu

**Affiliations:** ^1^Beijing Advance Innovation Center for Food Nutrition and Human Health, Beijing Laboratory of Food Quality and Safety, Beijing Engineering and Technology Research Center of Food Additives, Beijing Technology and Business University, Beijing, China; ^2^School of Control and Computer Engineering, North China Electric Power University, Beijing, China

**Keywords:** bacteriocin, co-culture, microorganism interaction, quorum sensing, response mechanism

## Abstract

Plantaricin is a kind of bacteriocin with broad-spectrum antibacterial activity on several food pathogens and spoilage microorganisms, showing potential in biopreservation applications. However, the low yield of plantaricin limits its industrialization. In this study, it was found that the co-culture of *Wickerhamomyces anomalus* Y-5 and *Lactiplantibacillus paraplantarum* RX-8 could enhance plantaricin production. To investigate the response of *L. paraplantarum* RX-8 facing *W. anomalus* Y-5 and understand the mechanisms activated when increasing plantaricin yield, comparative transcriptomic and proteomic analyses of *L. paraplantarum* RX-8 were performed in mono-culture and co-culture. The results showed that different genes and proteins in the phosphotransferase system (PTS) were improved and enhanced the uptake of certain sugars; the key enzyme activity in glycolysis was increased with the promotion of energy production; arginine biosynthesis was downregulated to increase glutamate mechanism and then promoted plantaricin yield; and the expression of several genes/proteins related to purine metabolism was downregulated and those related to pyrimidine metabolism was upregulated. Meanwhile, the increase of plantaricin synthesis by upregulation of *plnABCDEF* cluster expression under co-culture indicated that the PlnA-mediated quorum sensing (QS) system took part in the response mechanism of *L. paraplantarum* RX-8. However, the absence of AI-2 did not influence the inducing effect on plantaricin production. Mannose, galactose, and glutamate were critical metabolites and significantly simulate plantaricin production (*p* < 0.05). In summary, the findings provided new insights into the interaction between bacteriocin-inducing and bacteriocin-producing microorganisms, which may serve as a basis for further research into the detailed mechanism.

## Introduction

Bacteriocins are proteins or peptides that display antibacterial activity against various spoilage and pathogenic bacteria (Bu et al., 2021b). Bacteriocins from lactic acid bacteria (LAB) are regarded as safe, natural antimicrobials for food biological preservatives (Bu et al., 2021a). However, the manufacturing and secretion of bacteriocin have high metabolic costs; therefore, bacteria have developed regulatory mechanisms that relay quorum sensing (QS) signals to generate bacteriocins only upon necessity (Kern et al., 2021). As they lack competitors, bacteriocin production is low and may be gradually lost in ideal laboratory conditions. To increase bacteriocin production, it is useful to add certain microorganisms to induce bacteriocin-producing bacteria to produce more bacteriocins. This approach uses the interaction between different strains in mixed culture and was widespread in the area of natural bacteriocin production (Chanos and Mygind, 2016).

Plantaricin, a class IIb bacteriocin produced by *Lactiplantibacillus plantarum*, is often induced by the bacteria–bacteria interactions. The mechanism of induction regulation behind plantaricin production under co-culturing with other bacteria is associated with QS, the regulation of specific genes in a bacterial population. Specifically, it was reported that the plantaricin production of *L. plantarum* NC8 by co-culture is mediated by the three-component regulatory system, compounded by an autoinduction factor (PlNC8IF), the histidine kinase (PlNC8HK), and the response regulator (RR; PlnD) (Maldonado-Barragán et al., 2013). Furthermore, the interspecies QS molecule AI-2 and its related gene *LuxS* both increased in *L. plantarum* DC400 under co-culture (Calasso et al., 2013). Meanwhile, the two QS signals (PlnA and AI-2) independently participate in the plantaricin induction mechanism of the co-culture system (*L. paraplantarum* RX-8 and *Bacillus subtilis* BS-15) (Liu et al., 2022).

However, the inducing mechanism of bacteriocin production when bacteriocin-producing strains co-culture with fungi remains unknown, especially for plantaricin. It has been reported that undissociated lactate and a low pH strongly inhibit the growth of *Lactococcus lactis* and lead to a low yield of nisin, while *Kluyveromyces marxianus* MS1 (Wardani et al., 2006), *Yarrowia lipolytica* ATCC 18942 (Ariana and Hamedi, 2017), and *Saccharomyces cerevisiae* W303-1A (Liu et al., 2021) could increase nisin production by consuming lactate. However, research involving a detailed mechanism at the gene level is still inadequate. Unlike that in bacteria, the QS system in fungi has different models and the bacteria–fungi interaction has yet to be studied in detail. A deeper understanding of interaction networks between microbes, including bacteria–bacteria, bacteria–fungi, and fungi–fungi, will help in the exploration of the induction mechanism of yeast, which has an inducing effect on bacteriocin production, and provide a new method for control.

Plantaricin RX-8 produced by *L. paraplantarum* RX-8 was proven to exhibit broad-spectrum antibacterial activity, and the yield of plantaricin RX-8 was improved by *Wickerhamomyces anomalus* Y-5. In this study, interactions between *L. paraplantarum* RX-8 and *W. anomalus* Y-5, the pH, the plantaricin production, and the transcription levels of related bacteriocin biosynthesis genes from 4 to 32 h in co-culture/mono-culture were investigated. Furthermore, transcriptomic and proteomic technologies were used to compare the difference in gene and protein expression between the *L. paraplantarum* RX-8 cells under a co-culture and mono-culture.

## Methods and materials

### 2.1. Co-culture experiment

*L. paraplantarum* RX-8 (CGMCC 20852) was isolated from the traditional pickle in Chengdu, Sichuan Province, China. *W. anomalus* Y-5 was isolated from fermented grains of Chinese liquor and stored in the food quality and safety laboratory at Beijing Technology and Business University (Beijing, China). In brief, *L. paraplantarum* RX-8 and *W. anomalus* Y-5 were cultured overnight in De Man–Rogosa–Sharp (MRS) medium at 37°C and Yeast Peptone Dextrose (YPD) medium at 30°C under aerobic conditions, respectively. Then, 1 ml of *L. paraplantarum* RX-8 and *W. anomalus* Y-5 were singly inoculated into 100 ml of MRS liquid media for 24 h at 37°C as mono-cultured samples. Then, 1 ml of *L. paraplantarum* RX-8 and *W. anomalus* Y-5 were simultaneously inoculated into 100 ml of MRS liquid media for 24 h at 37°C as co-cultured samples.

### 2.2. Determination of viable cells and pH

The viable cells were counted every 4 h from 0 to 32 h, and the assay of viable cell counts was carried out according to the method of Zhang et al. (2021). At the same fermentation time points, the cell-free supernatants (CFSs) were obtained by removing cells through centrifugation (10,000 rpm, 10 min, 4°C). Then, the pH value of the CFSs was measured with a pH meter (PH400, Alalis, USA).

### 2.3. Plantaricin production assay

The yield of plantaricin in mono-culture/co-culture was assayed through agar well diffusion. After every 4 h (4–32 h) fermentation, the CFSs were obtained by centrifugation at 10,000 rpm for 10 min at 4°C; then, the pH of CFSs was adjusted to 6.0 with 1 mol/L NaOH, and these CFS samples were concentrated by vacuum centrifugal concentration (1,500 rpm, 45°C, 5 h). Wells of 6 mm were loaded with 100 μl of 5-fold concentrated plantaricin in the agar, which was inoculated at about 10^7^ CFU *Listeria monocytogenes* 35152. The plates were stored at 4°C overnight to allow for the plantaricin to completely diffuse; then, these plates were cultured for 8 h at 37°C. The bacteriocin activity was expressed in arbitrary units (AU/ml), which are represented as the reciprocal of the highest dilution, showing distinct zones of inhibition, and calculated according to the equation:


$$\begin{array}{l} Bacteriocin\;activity\left(\frac{AU}{mL}\right)\;=\frac{a^{n}}{b\times c} \end{array}$$

where a is 2 (dilution factor), n is the reciprocal of the highest dilution that resulted in inhibition of the indicator strain, b is 100 μl (sample volume in each well), and c is 5 (sample concentration fold). Furthermore, the relative bacteriocin activity was defined as the ratio of bacteriocin activity in co-culture to that in mono-culture. This was used to evaluate the influence of *W. anomalus* Y-5 on plantaricin production in co-culture.

To verify the role of bacteriocin in the competition of bacteria and yeast, the inhibiting effect of plantaricin on *W. anomalus* Y-5 was determined according to Section 2.3.

### 2.4. RT-qPCR analysis of plantaricin locus and AI-2 synthesis-related gene

To determine the expression level of plantaricin biosynthesis gene clusters (*plnABCDEF*) and AI-2 synthesis-related genes (*pfs* and *luxS*), cells of *L. paraplantarum* RX-8 were harvested through centrifugation (10,000 rpm, 2 min, 4°C) at every 4 h in mono-culture/co-culture (4–32 h). RNA was extracted from cells using the RNAprep Pure Cell/Bacteria Kit according to the manufacturer's instructions (DP430, Tiangen Biotech, China). The extracted RNA was reverse-transcribed into cDNA using the FastQuant RT Kit (with gDNase) (KR116, Tiangen Biotech, China). The transcriptional levels of *plnABCDEF, pfs*, and *luxS* were realized by CFX96 Real-Time PCR Detection System (Bio-Rad, Hercules, CA, USA) using SYBR Green PCR master mix (FP205, Tiangen Biotech, China). The reaction mixture consists of 10 μl 2×SuperReal PreMix Plus, 0.6 μl each of the forward and reverse primer, 1.0 μl cDNA (100 ng), and 7.8 μl nuclease-free H_2_O. The amplification program was performed at 95°C for 15 min, followed by 39 cycles of 95°C for 10 s, 55°C for 20 s, and 72°C for 30 s. Primers were designed based on the genome sequence of *L. paraplantarum* RX-8 and synthesized by Beijing Genomics Institute (Table 1). 16S rRNA genes were used as the reference gene. The relative expression levels of target genes at each sampling point for *L. paraplantarum* RX-8 in mono-culture/co-culture were calculated by the ΔΔCt method.

**Table 1:** Primer sequences used for RT-qPCR.

**Primer**	**Sequence (5^′^-3^′^)**
*16S rRNA*-F	GCATTAAGCATTCCGCCTGG
*16S rRNA*-R	ACCTGTATCCATGTCCCCGA
RT-*plnA*-F	CGCTGCGCTTAAGTTAATGT
RT-*plnA*-R	TTTTGAGGTACGCTGGGATT
RT-*plnB*-F	CGATACACAGGCTCGTTTGA
RT-*plnB*-R	GCCCAAGCATCAAAACAAAT
RT-*plnC*-F	GGATTGCACCGTTGGATTAT
RT-*plnC*-R	AGAAACGCGTTCCGATTTTA
RT-*plnD*-F	GTTGCAACGGATGATCAAAA
RT-*plnD*-R	ATAATCCAACGGTGCAATCC
RT-*plnE*-F	TTGAGAAGTTACAATATTCCAGGTTG
RT-*plnE*-R	CCCCTAATATTCAAAATACCACGA
RT-*plnF*-F	TGCTATTTCAGGTGGCGTTT
RT-*plnF*-R	GACAGCGCTAATGACCCAAT
RT-*luxS*-F	CGGATGGATGGCGTGATTGACTG
RT-*luxS*-R	CTTAGCAACTTCAACGGTGTCATGTTC
RT-*pfs*-F	GAATTATTTGTGCGATGGAAGAAGA
RT-*pfs*-R	CGCAAATAATGCAAGTAACATCGCT

### 2.5. Transcriptomic analysis

#### 2.5.1. RNA extraction, library construction, and transcriptome sequencing

Cell samples of *L. paraplantarum* RX-8 were obtained in co-culture and mono-culture at 24 h by centrifugation (10,000 rpm, 10 min, 4°C). After removing the supernatant, cells were immediately cleaned by phosphate buffered saline (PBS) two times, frozen in liquid nitrogen for 15 min, and then stored at −80°C.

Total RNA extractions were performed using Magen HiPure Universal RNA Kit (Magen, China). RNA concentration and purity were measured using Qubit 3.0 (Thermo Fisher Scientific, MA, USA) and Nanodrop One (Thermo Fisher Scientific, MA, USA), and integrity was confirmed using the Agilent 4200 system (Agilent Technologies, Waldbron, Germany). The RNA samples with an RNA integrity number (RIN) >6.5, and 280/260 ratios >1.5 were further used for RNA-sequencing purposes. Libraries were generated from three replicates using NEB Next^®^Ultra™ Directional RNA Library Prep Kit for Illumina^®^ (New England Biolabs, MA, USA). From these libraries, the PE150 reads were produced with the Illumina Novaseq6000 platform by Guangdong Magigene Biotechnology Co., Ltd. (Guangzhou, China). All raw RNA-Seq data were submitted to NCBI under BioProject PRJNA901346.

#### 2.5.2. Sequencing data processing

The raw reads were trimmed using Trimmomatic (version 0.36) to obtain the qualified reads (Bolger et al., 2014). Then, the rRNA sequences were removed in the qualified reads mapping with NCBI Rfam datasets using Bowtie2 (version 2.33) (Langdon, 2015). Initially, we mapped the mRNA reads of these samples to the reference genome *L. plantarum* WCFS1 downloaded from NCBI, which was the strain that was most closely related to *L. paraplantarum* RX-8, but the acquired alignment rate was mostly low at around 50% and limited to transcriptomic analysis. Therefore, to obtain a higher mapping rate, we assembled the draft genome of *L. paraplantarum* RX-8 using Unicycler (version 0.4.7) to assemble the combined Illumina NovaSeq PE150 data and Nanopore PromethION data (Wick et al., 2017). The new reference genome was uploaded to the NCBI shared database for research retrieval in NCBI GenBank CP111117-CP111119. Eventually, the transcriptomic data were mapped to the new reference genome using Hisat2 (version 2.1.0) (Kim et al., 2019), and the mapping rate was improved to above 90%. Read counts per genome assembly annotated transcript were calculated using HTseq (version 0.9.1) (Anders et al., 2015).

#### 2.5.3. Differential expression genes detection

The differentially expressed genes (DEGs) between groups (RY and R, three biological replicates) were identified from all genes according to the expression levels of the transcripts, based on the statistics of the fragments per kilobase of each reading, per million mapped reads (FPKM). The DEGs were detected using edgeR (version 3.16.5) with default parameters (Robinson et al., 2010). We then used Benjamini and Hochberg's correction approach to adjust the *p*-values to control the false discovery rate (FDR). The genes were regarded as DEGs if they satisfied the threshold with *FDR* ≤ 0.05 and |*Log*_2_*FC*|≥1. These genes were used for further enrichment analysis.

#### 2.5.4. Enrichment analysis

The enrichment analyses of DEGs with Gene Ontology (GO) and Kyoto Encyclopedia of Genes and Genomes (KEGG) databases were implemented using clusterProfiler (version 3.4.4), in which gene length bias was corrected. The GO terms and KEGG pathways with FDR ≤ 0.05 were regarded as candidates for enrichment functional annotation *via* the DEGs.

### 2.6. Proteomic analysis

#### 2.6.1. Cytosolic protein extraction and in-solution protein digestion

Cell samples of *L. paraplantarum* RX-8 in co-culture and mono-culture were collected at 24 h according to Section 2.5.1. The protein was extracted by using a lysis buffer (8 M urea, 50 mM Tris8.0, 1% NP40, 1% sodium deoxycholate, 5 mM dithiothreitol (DTT), 2 mM EDTA, 30 mM nicotinamide, and 3 μm trichostatin A), and, after sonication on ice, the total protein concentration of the supernatant, which was obtained by centrifugation (20,000 rpm, 10 min, 4°C), was determined by using a BCA Protein Assay kit. The protein sample was reduced by DTT (5 mM, 45 min, 30°C), later alkylated with 30 mM iodoacetamide (30 mM, 1 h, RT) in darkness, and then precipitated with ice-cold acetone. After being washed thrice with acetone, the precipitate was suspended in 0.1 M triethylammonium bicarbonate (TEAB) and digested with trypsin (1/25 protein mass, Promega) for 12 h at 37°C. Finally, the reaction was ended with 1% trifluoroacetic acid (TFA), and the resulting peptide was desalted with Strata X C18 SPE column (Phenomenex, Torrance, CA, USA) and vacuum-dried in Scanvac maxi-beta (Labogene, Alleroed, Denmark).

#### 2.6.2. TMT labeling and HPLC fractionation

After reduction and alkylation, six digested peptide samples (RY and R, three biological replicates, 100 μg/sample) were transferred to the TMT Reagent vial (Thermo Fisher Scientific, 90066) and incubated for 2 h at room temperature. Then, 8 μl of 5% hydroxylamine was added to the mixture and incubated for 15 min to quench the reaction. Finally, the labeled samples were fractionated into 15 fractions by XBridge Shield C18 RP column (Waters, Milford, MA, USA) in an LC20AD HPLC system (Shimadzu, Kyoto, Japan) for proteomic analysis.

#### 2.6.3. LC-MS analysis

The sample was loaded to the column equilibrated with buffers A (0.1% formic acid in water) and B (0.1% formic acid in acetonitrile). The Acclaim PepMap 100 C18 trap column (75 μm × 2 cm, Dionex) was equilibrated with liquid A by Ultimate 3000 nanoUPLC (Dionex), and the sample was eluted onto an Acclaim PepMap RSLC C18 analytical column (75 μm × 25 cm, Dionex) at a flow rate of 300 nl/min. The liquid-phase gradient was given as follows: 0–6 min, where the linear gradient of buffer B was from 2 to 10%; 7–51 min, where the linear gradient of buffer B was from 10 to 20%; 51–53 min, where the linear gradient of buffer B was from 20 to 80%; 53–57 min, where the linear gradient of buffer B solution was held at 80%.

Mass analysis was performed by nano-spray ionization-mass spectrometry (NSI-MS) and Q-Exactive HF mass spectrometry (Thermo Scientific). The intact peptide was detected by the Orbitrap, the scanning range was set to 250–1,500 m/z, the automatic gain control (AGC) target was 3E6, the resolution was 70,000, the max injection time (IT) was 250 ms, and the dynamic exclusion time was 15 s. The peptide was selected and fragmented for MS/MS using 28% NCE; ion fragments were detected in the Orbitrap, the resolution was 17,500, AGC was 1E5 or 5E4, and the maximum IT was 100 or 200 ms. LC-MS was performed by Micrometer Biotech (Hangzhou, China).

#### 2.6.4. Protein database search and protein quantification

To improve the accuracy of protein identification, RNA-Seq data as mentioned earlier were used to construct specific reference protein databases using DIAMOND (version 2.0.15) (Buchfink et al., 2015). The MS files were searched against the sample-specific *L. paraplantarum* RX-8 database using MaxQuant (version 1.5.2.8) (Tyanova et al., 2016). The mass error of precursor and fragment ions was set as 10 ppm and 0.02 Da, respectively. Trypsin was selected for enzyme specificity and two missed cleavages were allowed. Carbamidomethylation on Cys, TMT 6-plex tag of Lys, and peptide N-terminal was specified as a fixed modification. The variable modifications were oxidation on Met and TMT 6-plex tag on Tyr. FDR was estimated by reverse-decoy strategy, percolator algorithm, and peptide–spectrum match (PSM). *P* < 0.05 and *e* < 0.05 were used to calculate the results. For quantitation, a protein must have at least two unique peptides with the above identity. The protein ratio type was median, and the normalization method was median. All the raw files were uploaded to PRIDE with the accession number PXD038315.

#### 2.6.5. Bioinformatics analysis

The proteins identified as differentially expressed proteins (DEPs) should satisfy the threshold of *p* ≤ 0.05 and |*Fold Change*|≥1.3. The enrichment analysis of DEPs used the clusterProfiler (version 3.4.4) for GO function annotation and KEGG pathways (Yu et al., 2012). The GO terms and KEGG pathways were regarded as significant protein enrichment annotations using the DEPs.

### 2.7. Key differential genes verification

To validate the transcriptomic and proteomic data, RT-qPCR was employed to measure the expression of selected genes in mRNA, according to Section 2.4. The primers for selected genes were synthesized by the Beijing Genomics Institute, and the sequences are listed in Table 2.

**Table 2:** Primers used for key differential genes verification.

**Primer**	**Sequence (5^′^-3^′^)**
*16S rRNA*-F	GCATTAAGCATTCCGCCTGG
*16S rRNA*-R	ACCTGTATCCATGTCCCCGA
RT-*oppA*-F	ATACGACGATGCTGTGAAGAAG
RT-*oppA*-R	GTTGCTGAGGCTTGTTGATAGA
RT-*agrA*-F	GCCATTGTCAATCGCCGTAA
RT-*agrA*-R	TTAGATGATGGTGAACTGGAGTAC
RT-*pstS*-F	GGCACAAGGCTCTGGAACT
RT-*pstS*-R	TTAACGGTCTGTACGGACTGAT
RT-*glnA*-F	GGTATCGCTGACTTGCCATC
RT-*glnA*-R	GCTAACTTGCTGACGGTATGAT
RT-*agrH*-F	TGTCAGTTGGAGTAGTTATGAGTTC
RT-*agrH*-R	CCTTCATCACGGTCAATAGCC
RT-*purD*-F	CGGTCACATAATCTTCAATCAACAC
RT-*purD*-R	AGGCGGCAACGGCAATT

### 2.8. Key inducing metabolite verification

According to the results of transcriptomic and proteomic analyses, the carbohydrate substance (mannose, galactose, cellobiose, and D-ribose) was added to the mono-culture of *L. paraplantarum* RX-8 at the concentrations of 2, 20, and 200 mM. Meanwhile, the amino acid substance (arginine, cysteine, glutamate, and glutamine) was added to the mono-culture of *L. paraplantarum* RX-8 at the concentrations of 0.5, 1.25, and 2.5 g/L. After being cultured for 24 h at 37°C, the supernatants of each sample were collected for the plantaricin production assay. The mono-culture of *L. paraplantarum* RX-8 was used as a control.

### 2.9. Determination of AI-2 activity on inducing effect

To determine AI-2's role in inducing effect, AI-2 inhibitor D-ribose (0, 200, 300, and 400 mM) was added to the co-culture system; then, the plantaricin production of these samples at 24 h was detected according to Section 2.2, as described earlier. The plantaricin production in co-culture was used as the positive control, and that in mono-culture was used as the negative control.

The AI-2 activity was detected by the bioluminescence of *Vibrio harveyi* BB170. After overnight culture at 30°C, *V. harveyi* BB170 was diluted in a ratio of 1:5,000 with fresh AB medium. The CFSs of the above samples were adjusted to pH 7.0, then filtrated with a 0.22-μm sterile filter, and added to the diluted BB170 culture at the percentage of 10%. The mixture was incubated at 30°C for 4 h under aerobic conditions (180 rpm), and 200 μl of aliquots were added to white 96-well plates (Thermo, USA) to measure relative luminescence units (RLUs) using the Multi-Detection Plate Reader (SpectraMax i3, Molecular Devices, USA). The suspension of strain BB170 in AB medium (1:5,000) was used as a blank control.

## Results and discussion

### 3.1. Growth dynamics and bacteriocin production of *L. paraplantarum* RX-8 and *W. anomalus* Y-5 under co-culture

**Figure 1:**
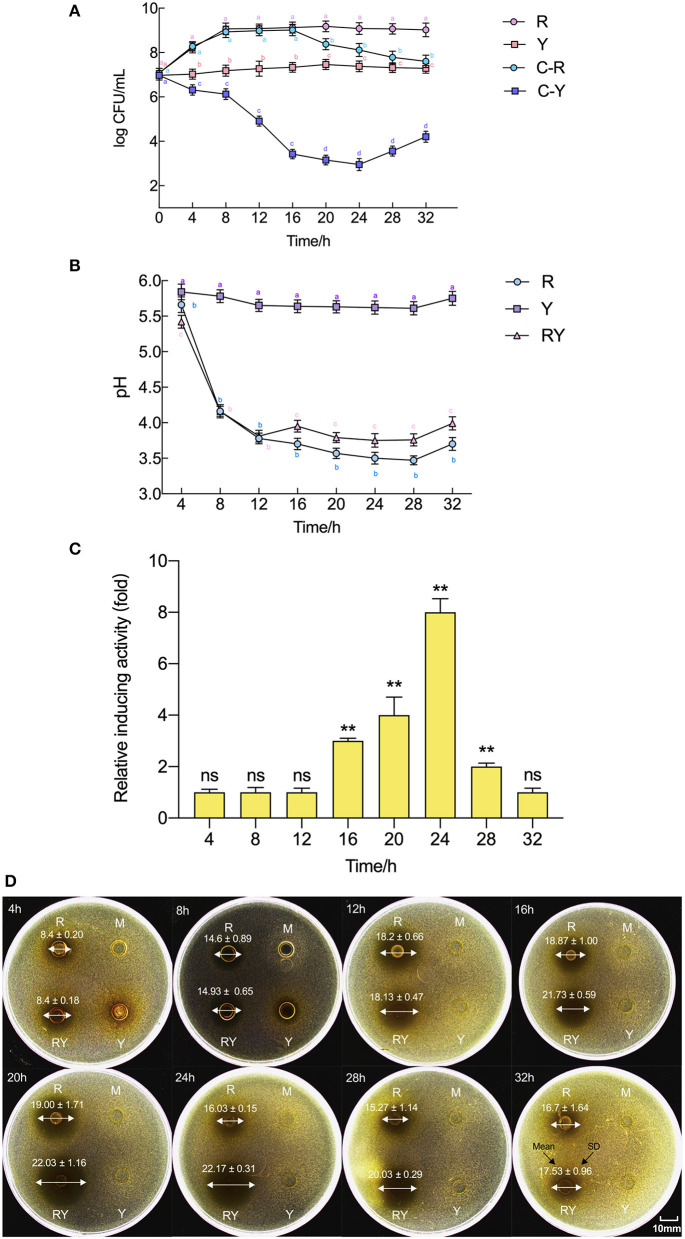
Evolution of pH, plantaricin production, and viable cell counts of *L. paraplantarum* RX-8/*W. anomalus* Y-5 in co-culture. **(A)** Indicates viable counts of *L. paraplantarum* RX-8/*W. anomalus* Y-5 at every 4 h from 4 to 32 h in mono-culture and co-culture, **(B)** indicates the pH value of CFS from mono-culture and co-culture at every 4 h from 4 to 32 h, **(C)** indicates the relative inducing activity in co-culture at every 4 h from 4 to 32 h compared with the mono-culture, **(D)** were images of inhibition zone of CFSs from mono-culture and co-culture against *L. monocytogenes* 35152. Mean diameter of inhibition zone (mm) ± standard deviation. R means mono-culture of *L. paraplantarum* RX-8 at 37°C in 4–32 h, Y means mono-culture of *W. anomalus* Y-5 at 37°C in 4–32 h, RY means bacteriocin-inducing co-culture of *L. paraplantarum* RX-8 and *W. anomalus* Y-5 at 37°C in 4–32 h, and M means blank control (MRS, pH 6.0). C-R means the viable cell count of *L. paraplantarum* RX-8 in co-culture, and C-Y means the viable cell count of *W. anomalus* Y-5 in co-culture. ns, not significant, *p < 0.05, **p < 0.01. Bars with no letter in common for each treatment are significantly different (*p* < 0.01).

The growths of *L. paraplantarum* RX-8/*W. anomalus* Y-5, the pH, and bacteriocin production in co-culture and every mono-culture from 4 to 32 h are shown in Figure 1. The viable cell numbers for the bacteria and yeast in co-culture were tested using different antibiotic susceptibilities of *L. paraplantarum* RX-8/*W. anomalus* Y-5. Up to 16 h, the growth rate of *L. paraplantarum* RX-8 was very consistent between the mono-culture and co-culture. However, a decline in *L. paraplantarum* RX-8 cell number was evident during 20–32 h. *W. anomalus* Y-5 grew poorly in liquid MRS media, as it lacked adequate oxygen and an appropriate temperature. The growth of *W. anomalus* Y-5 was also significantly reduced when *L. paraplantarum* RX-8 was present for 4–20 h, while *W. anomalus* Y-5 began to regrow over 24–32 h. The results of the antimicrobial assay show that plantaricin from *L. paraplantarum* RX-8 could not inhibit the growth of *W. anomalus* Y-5 (Supplementary Figure 1). A suitable pH for *W. anomalus* Y-5 growth was about 5.5. It was also reported that *W. anomalus* could not grow well under low pH conditions (Tian et al., 2020). The competitive effect of the two strains may be caused by nutrient competition and acid stress.

In the fermentation, *L. paraplantarum* RX-8 produced lactic acid and other organic acids, which rapidly decreased the pH value. The pH value of *L. paraplantarum* RX-8 under co-culture and mono-culture was similar from 4 to 12 h and rapidly declined from pH 5.7 to pH 3.8. The pH value in co-culture (pH 3.79–3.99) was slightly higher than that in mono-culture (pH 3.57–3.70) of *L. paraplantarum* RX-8 from 16 to 32 h. The pH value of *W. anomalus* Y-5 under mono-culture was maintained at 5.5–6.0. A reason for the increased pH in co-culture from 16 to 32 h may be that less organic acid is produced by *L. paraplantarum* RX-8, or it may be due to ammonia and proton production *via W. anomalus* Y-5.

Similarly, the bacteriocin production in co-culture did not increase until 16 h compared with that in mono-culture, then reached the highest point at 20 h, and stabilized in the 20–24 h period before declining at 28 h. Compared with the decrease in the population of *L. paraplantarum* RX-8, the bacteriocin activity of *L. paraplantarum* RX-8 in co-culture was promoted effectively. This suggests that the inducing effect of bacteriocin production is not due to an increase in cell number. Specifically, bacteriocin production at 24 h in co-culture finally reached 512 AU/ml and showed the most significant increase (8-fold) compared to that in mono-culture. Therefore, the sampling point (24 h) was selected for transcriptomic and proteomic analyses to determine the interaction between the two strains.

### 3.2. Effects of *W. anomalus* Y-5 on the transcription of plantaricin biosynthesis and AI-2 synthesis-related gene

**Figure 2:**
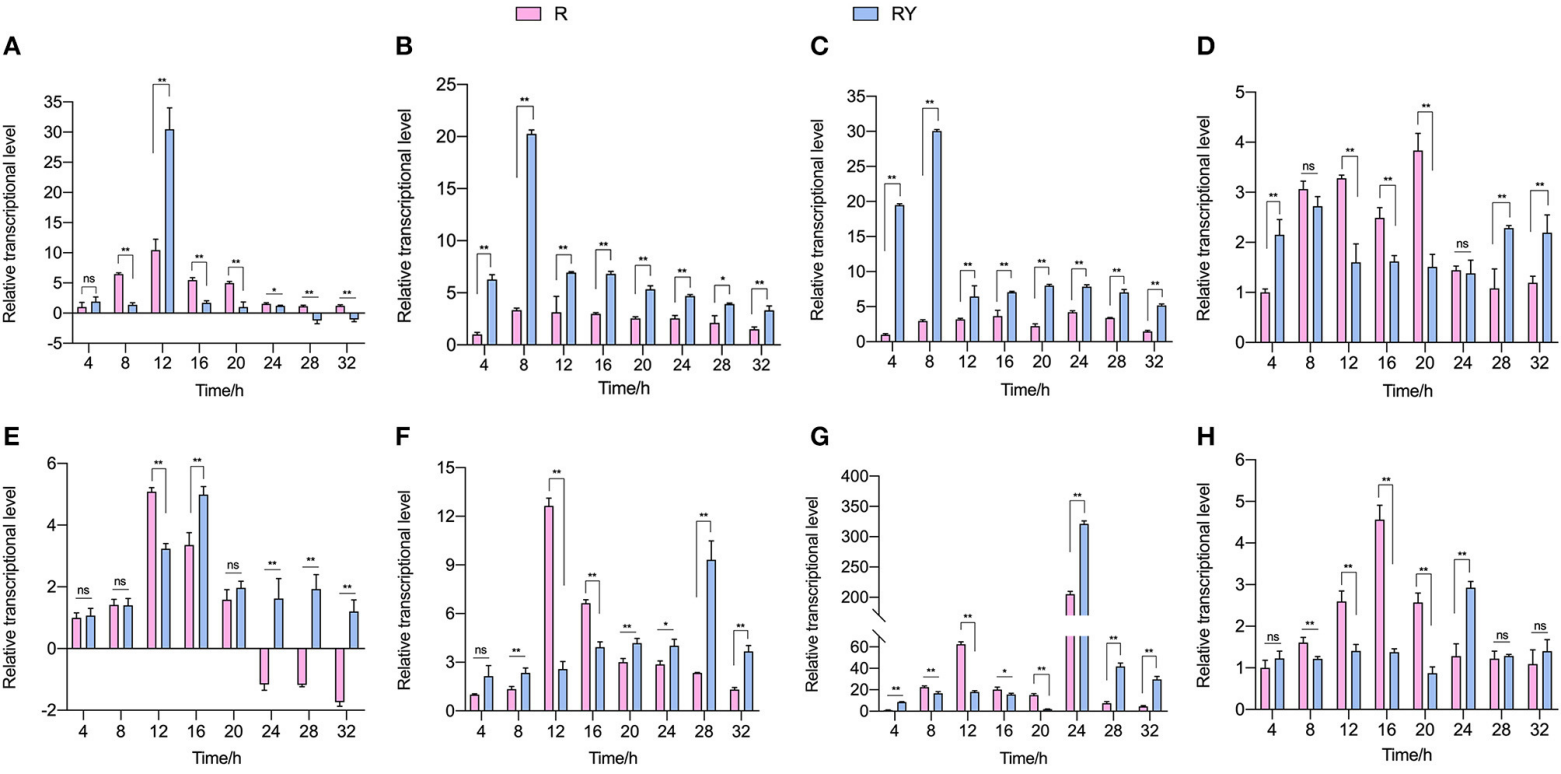
Transcriptional level of plantaricin biosynthesis (*plnABCDEF*; **A–F**) and AI-2 synthesis-related gene (*pfs* and *luxS*; **G, H**). R means mono-culture of *L. paraplantarum* RX-8 at 37°C in 4–32 h, and RY means bacteriocin-inducing co-culture of *L. paraplantarum* RX-8 and *W. anomalus* Y-5 at 37°C in 4–32 h. ns, not significant, *p < 0.05, **p < 0.01.

To explore *L. paraplantarum* RX-8's response to the inducing effect from *W. anomalus* Y-5, the plantaricin gene cluster *plnABCDEF* and AI-2 biosynthesis genes *pfs* and *luxS* in *L. paraplantarum* RX-8 were detected by RT-qPCR from 4 to 32 h. As shown in Figure 2, the transcriptional levels of the *L. paraplantarum* RX-8 genes in mono-culture (R) and co-culture (RY) increased over time and then decreased toward the end of their growth. The QS signal PlnA was encoded by *plnA*, which markedly increased in RY compared to that in sample R at 12 h, while the expression of *plnA* showed a decrease at 28 and 32 h in RY (Figure 2A). The reason for these phenomena is that the autoinducing peptide PlnA was secreted at an early stage before the production of bacteriocin. When the concentration of PlnA reached a critical threshold, it would activate the histidine protein kinase (HPK, encoded by *plnB*) on the cell membrane (Liu et al., 2022). However, the *plnB* trend was not consistent with the change of *plnA* in RY (Figure 2B). This indicates that some factors may induce an increase in *plnA*, and signal-transduction proteins may work with *plnB* on plantaricin production under co-culture. For instance, the overexpression of gene *Lp_2642*, belonging to the TetR family, and its regulatory factors could enhance plantaricin EF synthesis in *L. plantarum* 163, and the Lp_2642 protein can bind to the promoter sequence of the *plnA* gene *in vitro* experiments (Zhao et al., 2022). It was predicted that gene *lamK* (HPK) and *lamR* (RRs) would enhance the transcription of genes *plnB* and *plnD*, resulting in an increase in plantaricin EF production (Zhao et al., 2021). Then, the two RRs PlnC and PlnD were phosphorylated by HPK and bound to the promoters of *plnEF* to activate (by *plnC*) or repress (by *plnD*) the production of plantaricin EF (Straume et al., 2009). The transcriptional level of *plnC* in RY was higher than that in R over 4–32 h, and the transcriptional level of *plnD* in RY was lower than that in R over 12–20 h (Figures 2C, D). As for *plnEF*, the transcription expressions of *plnE* and *plnF* in RY were both obviously higher than those in R over 20–32 h, which was consistent with the changes in the plantaricin production (Figures 2E, F). The maximum transcriptional level of *plnE* in R was reached at 12 h; then, it was downregulated from 24 to 32 h. However, the expression of *plnE* in RY was the highest at 16 h, and it remained upregulated at 24–32 h. The maximum transcriptional level of *plnF* in R was reached at 12 h, while it reached the maximum point at 28 h in RY. For the gene of AI-2 biosynthesis' key enzymes, the transcriptional level of *pfs* and *luxS* decreased more in RY compared to R from 8 to 20 h, while they increased in RY at 24 and 28 h (Figures 2G, H). The AI-2 activity at 4–32 h showed the same trend in R and RY (Supplementary Figure 2). However, the effect of AI-2 on the induction process under co-culture is still unclear.

### 3.3. Transcriptomic and proteomic analyses of *L. paraplantarum* RX-8 in co-culture

**Figure 3:**
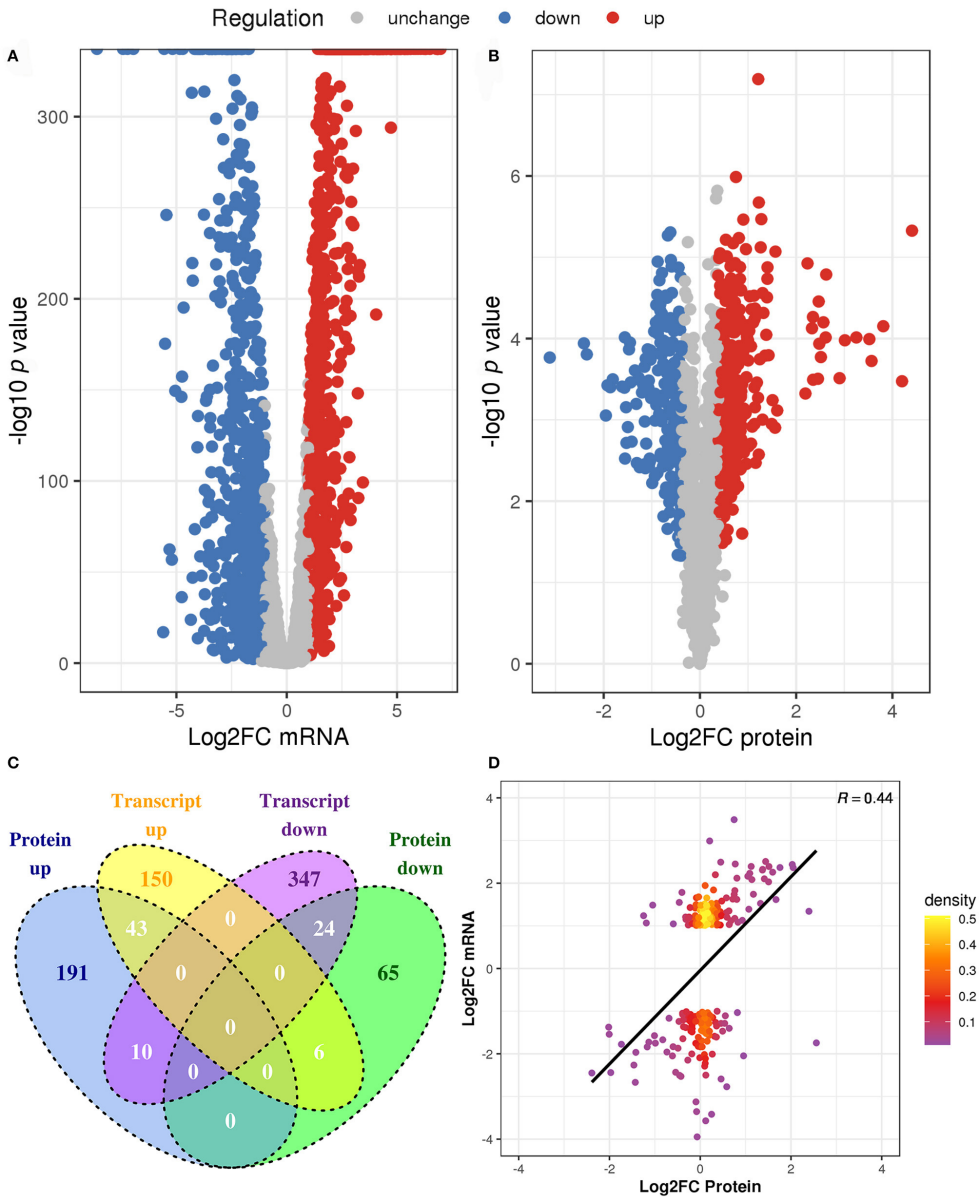
The changes of *L. paraplantarum* RX-8 under co-culture in transcriptional and protein levels. **(A)** Indicates volcano plots in the DEGs of *L. paraplantarum* RX-8 under co-culture. **(B)** Indicates volcano plots in the DEPs of *L. paraplantarum* RX-8 under co-culture. **(C)** Venn diagram illustrates the count of DEGs and DEPs coherence regulation, when comparing *L. paraplantarum* RX-8 in co-culture and mono-culture. **(D)** Correlation between DEGs and DEPs *L. paraplantarum* RX-8 under co-culture.

**Figure 4:**
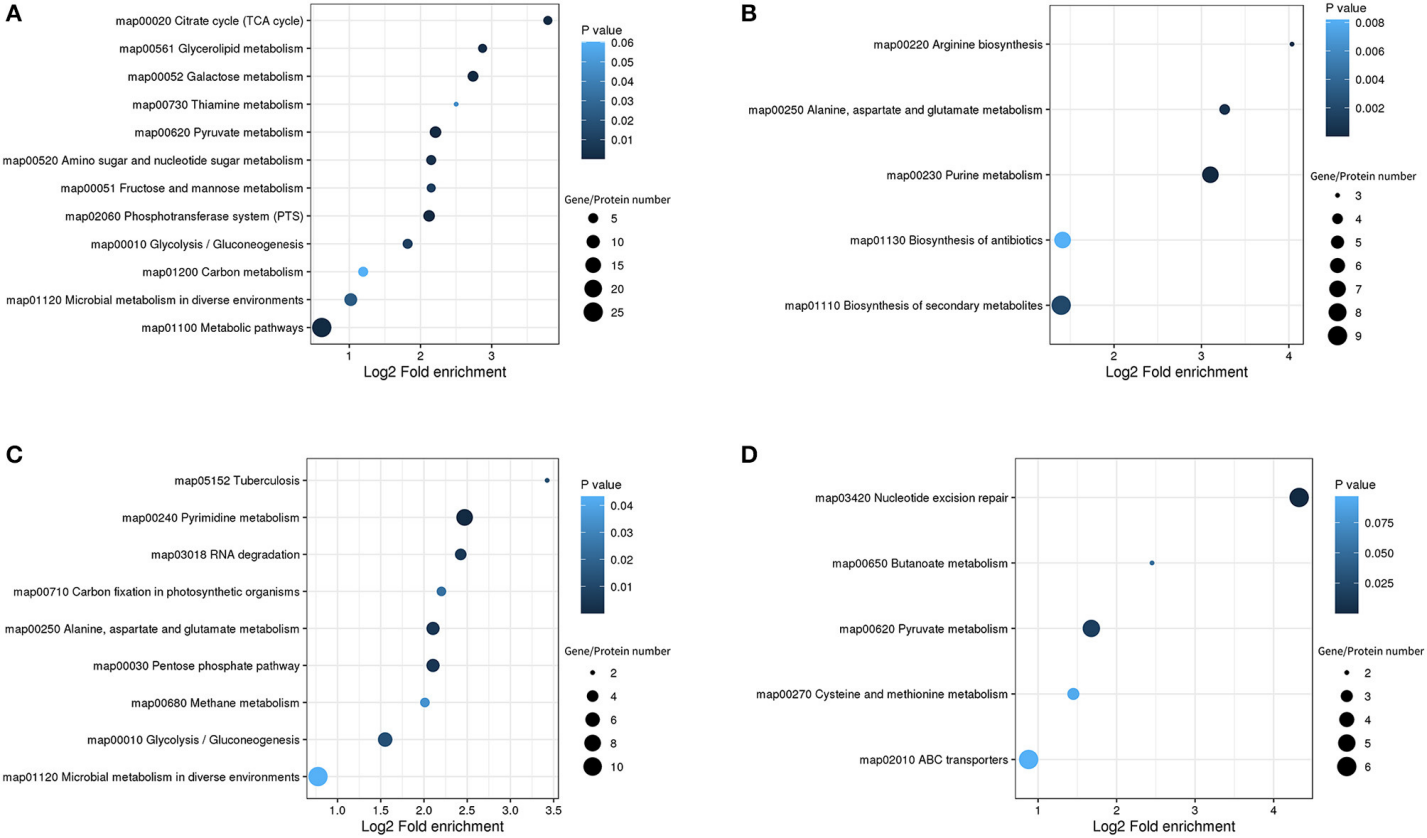
KEGG pathway enrichment bubble diagrams in the DEGs/DEPs of *L. paraplantarum* RX-8 under co-culture. **(A)** Indicates KEGG pathway enrichment of upregulated genes and upregulated proteins, **(B)** indicates KEGG pathway enrichment of downregulated genes and downregulated proteins, **(C)** indicates KEGG pathway enrichment of upregulated genes and unchanged proteins, and **(D)** indicates KEGG pathway enrichment of downregulated genes and unchanged proteins.

To identify the DEGs and DEPs of *L. paraplantarum* RX-8 in response to *W. anomalus* Y-5 under co-culture, the cells of *L. paraplantarum* RX-8 in co-culture and mono-culture were harvested at 24 h for transcriptomic and proteomic analyses. Due to a large amount of transcriptomic data and to reduce the false-positive rate, candidate genes of *L. paraplantarum* RX-8 involved in co-culture were chosen according to the following criteria: (1) more than 2-fold change after normalization; (2) Benjamini and Hochberg's correction approach was used to adjust the *p*-values; and (3) statistically significant level of *p* < 0.05. Finally, the transcription of 580 genes was detected, including 199 upregulated genes and 381 downregulated genes (Figure 3). In addition, iTRAQ was further performed to identify the DEPs of *L. paraplantarum* RX-8 in co-culture and mono-culture. Compared with the transcriptomic data, the amount of proteomic data was relatively small; therefore, FDR verification of the *p*-value was not needed. Compared to the mono-culture group, 339 DEPs (fold change ≥ 1.3, *p* < 0.05) were identified, including 244 upregulated proteins and 95 downregulated proteins (Figure 3). In the Venn diagrams, consistency between the significantly expressed genes and proteins was low. The correlation coefficient between mRNAs and proteins was 0.44 (Figure 3). This can be explained by some biological reasons, including weak ribosome-binding sites, regulatory proteins, codon usage bias, and the half-life difference between protein and mRNA, resulting in gene and protein expression levels that are not always perfectly correlated (Kumar et al., 2016). The 580 DEGs and 339 DEPs were subsequently applied to functional analysis in the functional enrichment of the KEGG pathway. After KEGG analysis, these pathways were remarkably upregulated under co-culture, which included the phosphotransferase system (PTS), glycolysis, galactose metabolism, and pyruvate metabolism (Figure 4). Other pathways were significantly downregulated under co-culture, which included purine metabolism and arginine biosynthesis (Figure 4).

#### 3.3.1 Carbohydrate transport and metabolism of *L. paraplantarum* RX-8 in co-culture

Compared with *L. paraplantarum* RX-8 in mono-culture, *L. paraplantarum* RX-8 in co-culture presented a much stronger carbohydrate utilization ability, which contributes to its great adaptability in competition with *W. anomalus* Y-5. In the process of carbohydrate transport and metabolism, the following metabolic pathways are involved in *L. paraplantarum* RX-8, with or without co-culture: PTS, fructose and mannose metabolism, galactose metabolism, glycolysis, TCA cycle, and amino sugar and nucleotide sugar metabolism.

The phosphotransferase system is responsible for the absorption of sugars, which depends on ATP and the 50 DEGs and 13 DEPs enriched in the PTS. In the PTS, the genes and proteins of mannose transport (*manXaYZ*) and galactitol transport (*gatABC*) are upregulated, while genes and proteins of fructose transport (*FruA/B*) are downregulated. In *Lactobacillus pentosus*, the mannose PTS EIIB participates in the inhibition of the fructose PTS (Chaillou et al., 2001). The mannose PTS in *L. paraplantarum* RX-8 may have a similar effect on inhibiting fructose transport (*FruA/B*). Aside from mannose, other carbohydrates, such as glucose, cellobiose, and maltose, could be transported by the mannose PTS. The sensing of exogenous AI-2 in *Streptococcus pneumoniae* is dependent on FruA; then, AI-2 promotes the transition of the pneumococcus from colonization to invasion by facilitating the utilization of galactose (Trappetti et al., 2017). In *Escherichia coli*, the internalization of AI-2 also depends on the PTS (Ha et al., 2018). AI-2 plays an important role in the inducing effect under a bacteria–bacteria co-culture (Chanos and Mygind, 2016; Liu et al., 2022), while AI-2's role in inducing this effect under fungi–bacteria co-culture needs further investigation. The proteins celA, celB, and celC, comprising the cellobiose transport system permease, take charge of the cellobiose utilization in *L. paraplantarum* RX-8, and are significantly upregulated, by 2.639-, 1.375-, and 2.71-fold, compared to that in mono-culture. However, the gene *celA* showed the opposite expression trend and the gene *celC* did not change. This indicates that post-transcriptional regulation may occur in *L. paraplantarum* RX-8. The gene *crr* encodes the sugar PTS EIIA component; when it is non-phosphorylated, Crr can inhibit the uptake of certain sugars, such as maltose, melibiose, lactose, and glycerol. However, when Crr is phosphorylated, it may activate adenylate cyclase (Saier and Roseman, 1976). The downregulation of the gene *crr* suggests that sugar uptake restrictions are lifted. Moreover, the mannose PTS EIIC acts as a target protein to confer class II bacteriocin sensitivity in *L. monocytogenes, Enterococcus faecalis*, and *Latilactobacillus sakei* (Kjos et al., 2009; Jeckelmann and Erni, 2020). The class IIa bacteriocin, immunity protein, and receptor proteins (mannose PTS EIIC and EIID) build up a tight complex to prevent pore formation and block cell death (Diep et al., 2007). However, the relationship between the mannose PTS and the immunity of class IIb bacteriocin produced by *L. plantarum* has not been reported to date (Kjos et al., 2010). Apart from its dual role in carbohydrate transport, the PTS is involved in the coupling of the sensory and regulatory mechanisms (Lengeler and Jahreis, 2009). The PTS transporter subunit IIB may be related to the effect of β-glucooligosaccharides on enhancing nisin production in *L. lactis* I2 (Lee et al., 2020). According to the results of transcriptomic and proteomic analyses, the PTS seems to be involved in the regulation of the response of *L. paraplantarum* to *W. anomalus* Y-5, leading to an increase in bacteriocin production.

**Figure 5:**
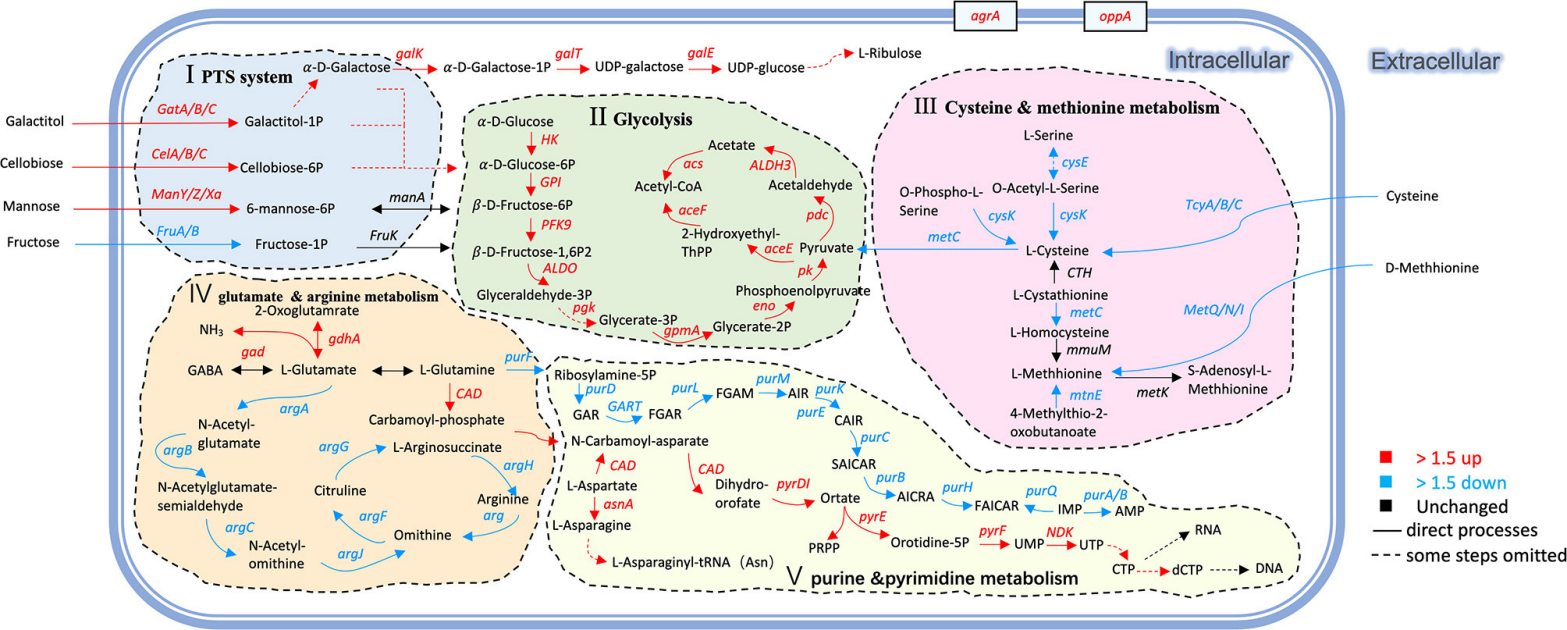
Changes of genes and proteins in *L. paraplantarum* RX-8 response to inducing effect from *W. anomalus* Y-5. The serial number (I, II, III, IV, V) indicates the response pathways of *L. paraplantarum* RX-8 under co-culture. (I) improving PTS and enhancing the uptake of certain sugars; (II) increasing the key enzyme activity in glycolysis, then promoting the energy production; (III) repressing the precursor of QS signal AI-2; (IV) decreasing arginine biosynthesis to increase glutamate mechanism, then promote plantaricin yield; (V) changing the expression of several genes/proteins related to purine metabolism (downregulation) and pyrimidine metabolism (upregulation). Pathways were constructed based on information provided in the KEGG database and previous studies.

Under co-culture conditions, the genes and proteins responsible for the utilization of cellobiose, lactose, galactose, fructose, sorbitol, galactitol, and ribose were also upregulated in *L. paraplantarum* RX-8. These sugars undergo glycolysis to pyruvate and ATP. Glucose-6-phosphate isomerase (GPI) was upregulated by 2.63-fold, which catalyzed the mutual conversion of glucose-6-phosphate and fructose-6-phosphate and helped sugars to enter the glycolysis pathway. The upregulated phosphoglycerate kinase (PGK), which directly generates ATP by phosphorylating the ADP, suggested that *L. paraplantarum* RX-8 produced more energy under co-culture. The pyruvate produced by glycolysis is a major metabolic intersection connecting the utilization and biosynthetic pathways of carbohydrates or amino acids; it linked the L-cysteine and carbon metabolism (Figure 5). After pyruvate dehydrogenase complex catalysis, pyruvate is oxidatively decarboxylated with the loss of one carbon and converted into acetyl-CoA, before entering the TCA cycle (Tian et al., 2022). The upregulation of DEPs in the TCA cycle enhances glucose oxidation and energy production. However, the increase in TCA decreased the transformation from phosphoenolpyruvate to aspartate, thereby weakening the serine synthesis. This leads to the downregulation of aspartate, serine, and L-cysteine metabolism.

#### 3.3.2. Amino acid metabolism of *L. paraplantarum* RX-8 in co-culture

Amino acids could serve as the materials in plantaricin synthesis. Aminoacyl-tRNA was responsible for the transportation of amino acids to ribosomes for incorporation into polypeptide chains of plantaricin. Under co-culture, the aspartate–ammonia ligase (*asnA*) and asparagine–tRNA ligase (*asnS*) increased by 2.02- and 2.35-fold, respectively. The aspartate–ammonia ligase catalyzed the production of L-asparagine; then, it was converted by asparaginyl–tRNA synthetase into L-asparaginyl–tRNA, which played a vital role in the translation of RNA into protein (Yeom et al., 2020). In addition, the enzymes that catalyzed the conversion of L-aspartate into fumarate were downregulated. The L-aspartate concentration in *Lactobacillus casei* increased when it faced acid resistance, and the transcriptional levels of *argG* and *argH* in *L. casei* increased with the decrease in pH (Wu et al., 2013). In our study, the pH of *L. paraplantarum* RX-8 in mono-culture was lower than that in co-culture, and a higher expression of *argG* and *argH* was detected in mono-culture.

At the same time, glutamate decarboxylase (*gad*), which was responsible for the mutual conversion of L-glutamate and γ-aminobutyrate (GABA), increased by 2.02-fold. The *gad* process converted L-glutamate into GABA, accompanied by the consumption of H^+^. This may explain why the pH of the co-culture was higher than that of the mono-culture of *L. paraplantarum* RX-8. Considering the upregulation of glutamate decarboxylase (*gdhA*), it is possible that *L. paraplantarum* RX-8 under co-culture produces more ammonia or proton consumption *via* glutamate decarboxylase (Teixeira et al., 2014). Moreover, the repression of GlnR could increase the expression of glutamine- and glutamate-metabolism-related genes (*glnP, glnQ, amtB, glnK, gltB*, and *gad*), promoting cell growth, acid resistance, and nisin yield (Miao et al., 2018). However, direct interactions between the anti41, an sRNA with a strong inhibitory effect on *glnR*, and the nisin gene cluster were not found (Miao et al., 2018). In addition, purine metabolism was weakened when the gene *purF* was downregulated, while pyrimidine metabolism was enhanced when the gene *CAD* was upregulated (Figure 5). The expression of carbamoyl–phosphate synthase (CAD) converted L-glutamine into carbamoyl phosphate, which is an intermediate in the biosynthesis of nucleotides through the orotate pathway (Namrak et al., 2022).

Meanwhile, the related arginine biosynthesis (*argACDJGH*) genes were downregulated, especially the genes controlling the transportation from glutamate to arginine (Figure 5). It was indicated that *L. paraplantarum* RX-8, under co-culture, led to the accumulation of glutamate by reducing arginine biosynthesis. The gene *metC*, which converted L-cysteine into L-methionine and was a precursor of S-ribosomal homocysteine (SRH), was downregulated. The gene *luxS*, a key enzyme, converted SRH into 4,5-dihydroxy-2,3-pentanedione (DPD). Then, DPD underwent spontaneous rearrangements to form AI-2. The downregulation of *luxS* influences the biosynthesis of AI-2, and a similar tendency was observed in co-culture AI-2 activity (Supplementary Figure 2). It was reported that the decrease in the L-methionine and L-cysteine cycling pathways was caused by the knockout of the *luxS* gene (Qian et al., 2022b). Similar results of changes in L-methionine- and L-cysteine-related genes were found in co-culture.

#### 3.3.3. Quorum sensing system of *L. paraplantarum* RX-8 in co-culture

Some genes related to the quorum-sensing system and ABC-transport system were upregulated in *L. paraplantarum* RX-8 under co-culture. OppA is an oligopeptide-binding protein that is responsible for peptides' capture (Kieliszek et al., 2021). Therefore, the strong upregulation of *oppA* suggested an increase in the transportation of oligopeptides. OppA could increase Ox-bile resistance in *L. salivarius* Ren and salt resistance in *Lactobacillus paracasei* ATCC 334 (Ma et al., 2018). It is possible that OppA helps *L. paraplantarum* RX-8 to survive in the presence of yeast. When PlnA or *Lactobacillus sanfranciscensis* DPPMA174 is co-cultured with *L. plantarum* DC400, OppA is upregulated compared with *L. plantarum* DC400 mono-culture (Calasso et al., 2013). Although *oppA* did not show a difference in the plantaricin Q7 biosynthesis of *L. plantarum* Q7, *oppD*-, and *oppF*-encoding intracellular ATPases were upregulated when plantaricin Q7 production increased and downregulated when plantaricin Q7 was hydrolyzed to support the nutrients needed to promote the growth of *L. plantarum* Q7 (Bu et al., 2021a). Moreover, the Opp system in *E. faecalis* and *Streptococcus mutans* has specific pheromone binding activity (Leonard et al., 1996; Li and Tian, 2012). It can be hypothesized that *L. plantarum* DC400 transports PlnA *via* the Opp system (Calasso et al., 2013). The upregulation of phosphate-inducible transport system (*pstSCAB*) suggested that the phosphate uptake was increased to meet the increase in the phosphorylated nucleotides and proteins, which control the biosynthesis of many secondary metabolites. In addition, fructose, galactose, or mannose in media could promote the expression of the *pstSCAB* operon and significantly increase the formation of PstS (Martín and Liras, 2021). In this result, the mannose transport system and galactose metabolism were also upregulated.

The production of plantaricin was regulated by the quorum-sensing system (encoded by *plnABCDEF* gene clusters) (Zhao et al., 2021; Liu et al., 2022). Although *plnABCDEF* gene clusters synthesizing bacteriocin did not show significant differences (|*Log*_2_*FC*|≥1, *p* < 0.05) in transcriptomic analysis, the *plnABCDEF* gene clusters were found to show 2.51-, 1.61-, 1.45-, 1.27-, 1.03-, and 1.41-fold changes at 24 h in transcriptomic analysis, and the results of RT-qPCR indicated that *W. anomalus* Y-5 stimulated plantaricin production *via plnABCDEF*. In the QS system, the gene *agrA*, an RR, was upregulated under co-culture. The Agr-mediated QS system (*agrBDCA*) might participate in commensal host–microbe interactions, especially *agrA*-encoded RR, which performs important functions in cell adhesion and biofilm formation in *L. plantarum* WCFS1 (Sturme et al., 2005). In addition, the expression of *agrA* was related to a global cellular response, such as the upregulated pyrimidine biosynthesis genes (*pyrB, pyrD, pyrF*, and *pyrR*) in *L. plantarum* WCFS1 (Sturme et al., 2005). Interestingly, similar results were also found for *L. paraplantarum* RX-8 in co-culture, suggesting that *agrA* controls pyrimidine biosynthesis under the interactions of *L. paraplantarum* RX-8 and *W. anomalus* Y-5. *agrBDCA* may influence plantaricin Q7 biosynthesis; specifically, *agrC* and *agrA* were upregulated in the synthesis of plantaricin Q7 (Bu et al., 2021a). The gene *agrB* of *L. paraplantarum* RX-8 was upregulated by 1.5-fold under co-culture. Protein AgrB also increased when *L. plantarum* DC400 was co-cultured with *L. plantarum* DPPMA20 or *L. sanfranciscensis* DPPMA174 (Calasso et al., 2013). This indicates that the *agrBCDA* quorum-sensing system might enhance plantaricin production when *L. paraplantarum* RX-8 is induced by *W. anomalus* Y-5.

#### 3.3.4. RNA degradation and nucleotide repair of *L. paraplantarum* RX-8 in co-culture

To repair DNA damage, there are different classes of DNA repair systems. The nucleotide excision repair (*uvrABCD*) system recognizes the damaged strand, removes it, and then fills the gap (Goosen and Moolenaar, 2001). The gene *uvrABCD* was downregulated, which indicates that there is less DNA synthesis or less DNA damage when *L. paraplantarum* RX-8 is under co-culture. It is important for a tightly regulated RNA metabolism to adapt to environmental change and utilize nutrients. RNA helicases could locally unwind double-stranded RNA, or they can clamp protein complexes to a substrate RNA in an ATP-dependent manner. Gene *cshA*-encoded RNA helicase was downregulated in *L. paraplantarum* RX-8 under co-culture. It was reported that the *cshA* mutant of *Staphylococcus aureus* SA564 led to increased stability in *agr* mRNA, and it was hypothesized that the *agrBDCA* mRNAs were unable to correctly degrade in the absence of *cshA* (Oun et al., 2013). This would account for the lower *cshA* and higher *agrA* expression in *L. paraplantarum* RX-8 under co-culture. GroEL could partially unfold the misfolded RNA or proteins in an ATP-dependent manner. DnaK is a major chaperone, and the binding and release of protein clients by DnaK are regulated by ATP's hydrolytic activity. The downregulation of *groEL* and *dnaK* may indicate that there is less RNA degradation and a more stable RNA in co-culture.

### 3.4. Transcription- and protein-level validation

**Figure 6:**
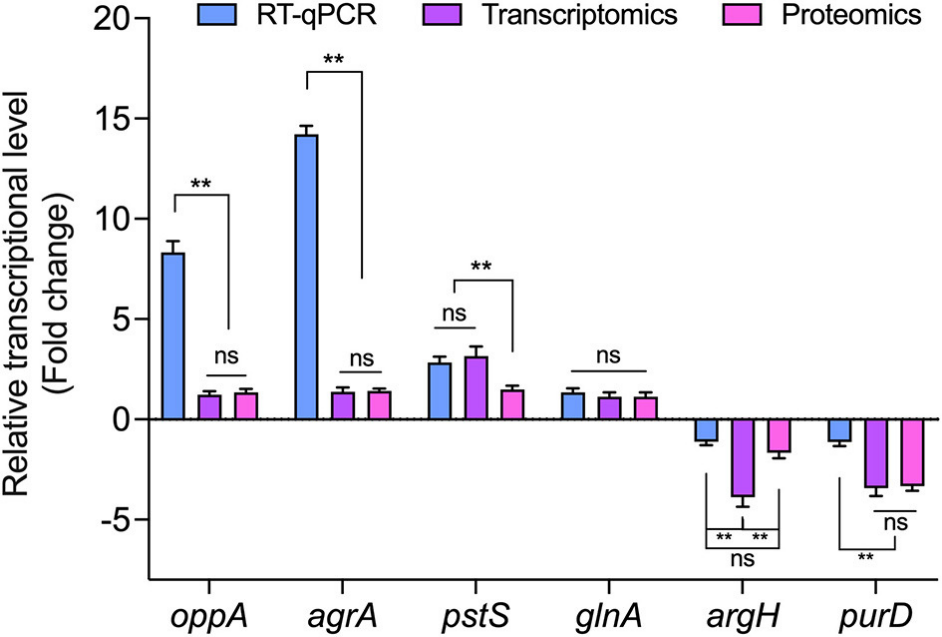
The relative expression of genes encoding DEPs in *L. paraplantarum* RX-8 under co-culture. ns, not significant, **p < 0.01.

Based on the results of transcriptomic and proteomic analyses, *oppA, agrA, pstS, glnA, argH*, and *purD* (four upregulated and two downregulated) were selected for validation by RT-qPCR. Although the value of the obtained fold-change was different between RT-qPCR and transcriptomic and proteomic data, the expressions of the tested genes were entirely consistent in the direction of regulation (Figure 6). The concordance between RT-qPCR and transcriptomic and proteomic data suggests high reliability in transcriptomic and proteomic data.

### 3.5. Inducing effects of key metabolites on plantaricin production

**Figure 7:**
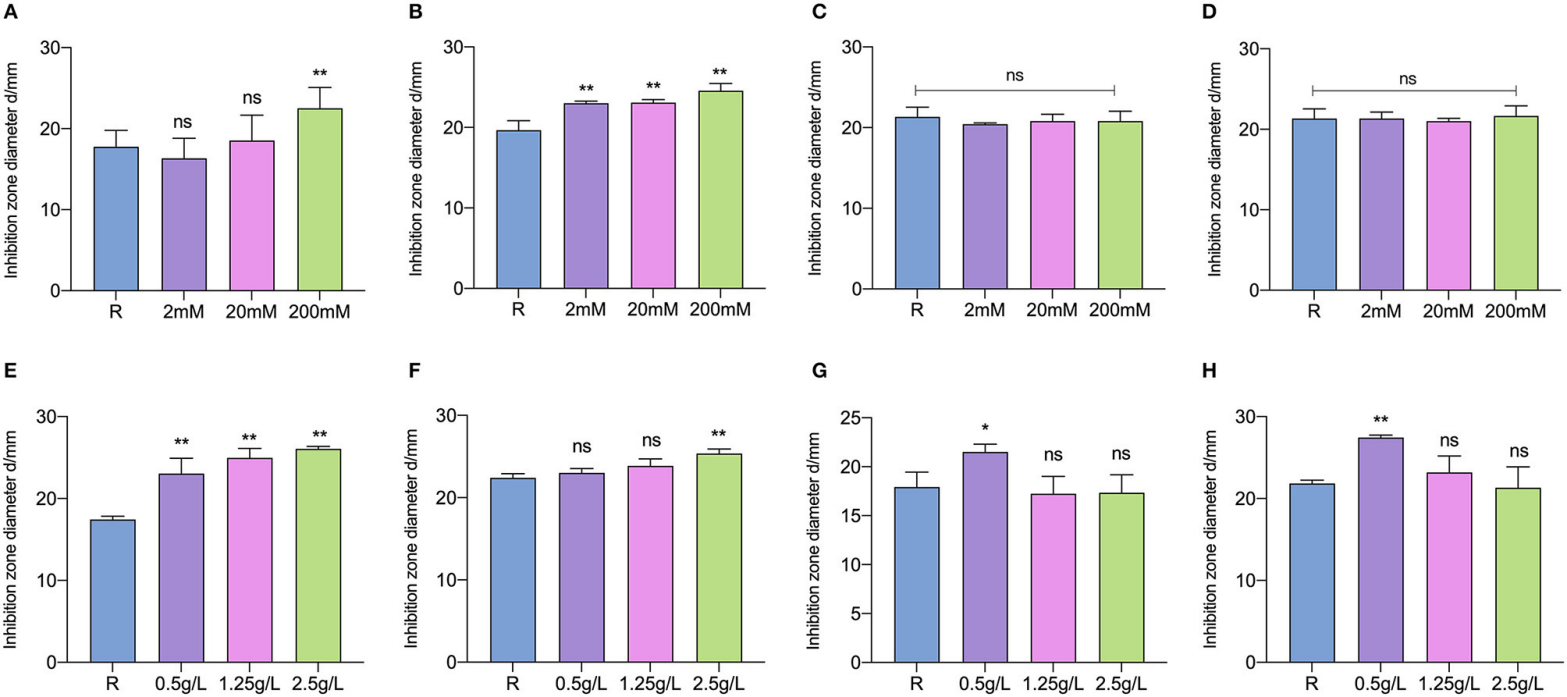
Inducing effects of different concentrations of galactose **(A)**, mannose **(B)**, cellobiose **(C)**, D-ribose **(D)**, arginine **(E)**, cysteine **(F)**, glutamate **(G)**, and glutamine **(H)** on plantaricin production. ns: not significant, *p < 0.05, **p < 0.01.

The effects of key-inducing metabolites on the plantaricin production of *L. paraplantarum* RX-8 were studied. It was found that the addition of galactose, mannose, arginine, cysteine, glutamate, and glutamine could induce plantaricin production, while cellobiose and D-ribose had little less effect on plantaricin production (Figure 7). It was indicated that galactose, mannose, arginine, cysteine, glutamate, and glutamine could be essential to the plantaricin production of *L. paraplantarum* RX-8. The production of plantaricin was significantly enhanced in mono-culture with the addition of 200 mM, but lower concentrations of galactose (2 and 20 mM) did not induce any effects on plantaricin yield (Figure 7A). The exogenous addition of mannose could increase the plantaricin synthesis at concentrations of 2, 20, and 200 mM (Figure 7B). The yield of plantaricin was strengthened at 24 h by adding arginine to mono-culture (Figure 7E). The addition of arginine, 2.5 g/L of cysteine, 0.5 g/L of glutamate, and 0.5 g/L of glutamine had significant effects on plantaricin production (Figures 7E, F, H), while cysteine, glutamate, and glutamine at other concentrations did not show a significant impact on plantaricin production.

Interestingly, the stimulus effects of glutamate and glutamine were not exhibited at higher concentrations. Considering the upregulation of related genes and proteins in galactose metabolism, this could because the significant induction that occurs at low concentrations of glutamate and glutamine makes them the most economic strategy for enhancing plantaricin production in *L. paraplantarum* RX-8. Bu et al. (2021b) reported that the plantaricin synthesis of *L. plantarum* Q7 was promoted by the exogenous addition of glutamate at 16 h, but plantaricin Q7 production was not stimulated by glutamate at 2, 6, and 12 h. Although *S. cerevisiae* could secrete different kinds of amino acids that are crucial to the survival of *L. plantarum*, the supplementing CDM35 medium with glutamine, threonine, phenylalanine, tryptophan, and serine, which were under the observed concentrations, had only a minimal impact on *L. plantarum* growth (Ponomarova et al., 2017). This is because the synergistic nature of multi-component cross-feeding was challenging to characterize. In addition, the nutritional competition was one of the reasons that *L. plantarum* SS-128 inhibits the growth of *Shewanella baltica* (Qian et al., 2022a). To further demonstrate the advantage of using *W. anomalus* Y-5 to induce bacteriocin production, metabolomics would be performed on quantified amino acids, sugar, and other metabolites secreted by *L. paraplantarum* RX-8 and *W. anomalus* Y-5. Then, the induction effect of the target metabolite at the observed concentrations in mono-culture would be analyzed.

### 3.6. The role of AI-2 activity in inducing effect

**Figure 8:**
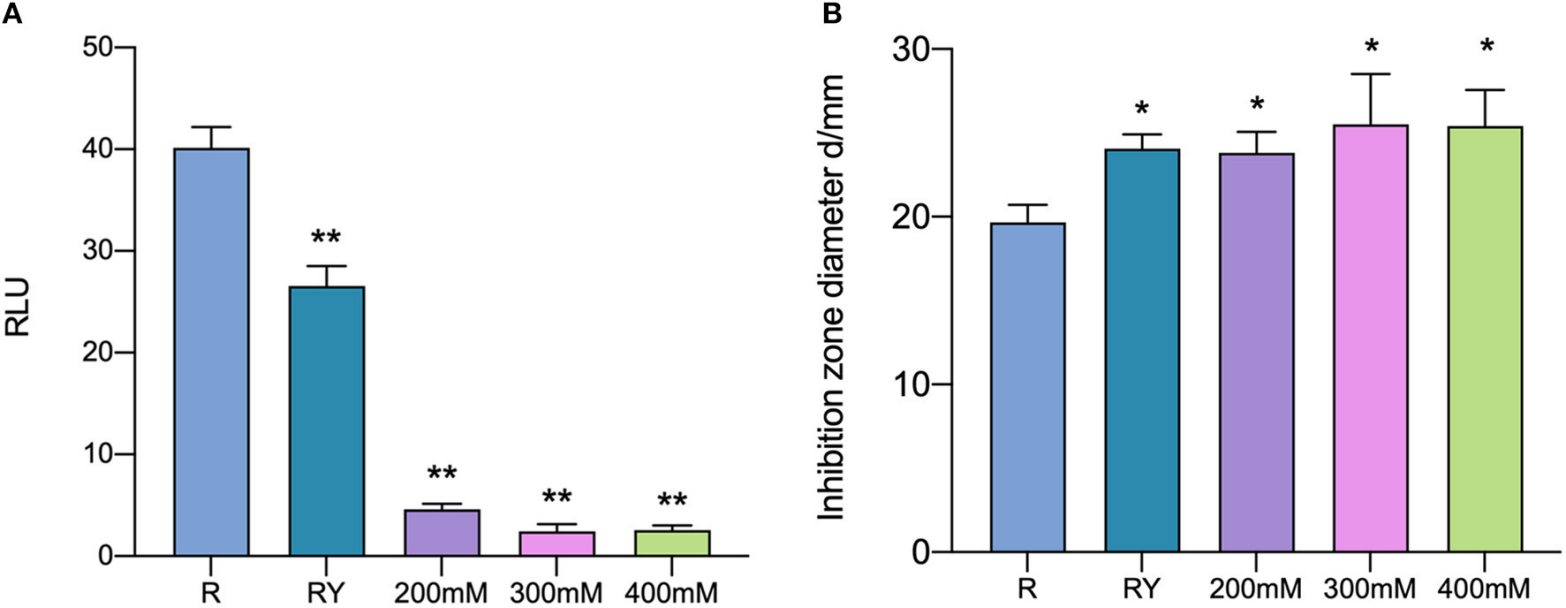
AI-2 activity **(A)** and the antibacterial activity **(B)** in co-culture with the addition of D-ribose. R means mono-culture of *L. paraplantarum* RX-8 at 37°C; RY means bacteriocin-inducing co-culture of *L. paraplantarum* RX-8 and *W. anomalus* Y-5 at 37°C as a positive control; 200/300/400 mM means co-culture of *L. paraplantarum* RX-8 and *W. anomalus* Y-5 at 37°C with 200, 300, and 400 mM D-ribose. *p < 0.05, **p < 0.01.

D-ribose, as a competitive inhibitor of AI-2, could cut the intracellular communication mediated by AI-2 among bacteria. AI-2 activity in co-culture was inhibited by D-ribose at all selected concentrations (Figure 8A). All samples whose AI-2 activity was cut still showed antibacterial activity and did not show a significant difference compared with co-culture samples without D-ribose (Figure 8B). It was suggested that AI-2 does not play an important role in inducing bacteriocin production when the bacteriocin-producing strain is co-cultured with *W. anomalus* Y-5.

## Conclusion

In this study, the plantaricin production and response mechanisms that occur when *L. paraplantarum* RX-8 is co-cultured with *W. anomalus* Y-5 were investigated. The results indicated that *W. anomalus* Y-5 could enhance plantaricin production by activating the expression of *plnABCDEF* cluster in *L. paraplantarum* RX-8. Then, the differences between *L. paraplantarum* RX-8 at transcriptomic and protein levels were analyzed under co-culture and mono-culture. The data showed that multiple metabolic pathways of *L. paraplantarum* RX-8 were significantly affected in the co-culture including (i) upregulation of the PTS in mannose, galactitol, and cellobiose transport, and downregulation of the PTS in fructose transport; (ii) modulation of the expression of several enzymes involved in glycolysis and other carbohydrate pathways; (iii) upregulation of key enzymes involved in glutamate metabolism and downregulation of multiple enzymes involved in aspartate, arginine, and cysteine metabolisms; (iv) modulation of the expression of several enzymes involved in the quorum-sensing system and ABC-transport system; and (v) upregulation of several enzymes involved in pyrimidine metabolism and downregulation of several enzymes involved in purine metabolism. In combination with the induction of metabolites' validation during plantaricin production and previous studies, we predicted that *L. paraplantarum* RX-8 mechanisms would occur in response to *W. anomalus* Y-5; that the changes in the PTS led to an increase in mannose and galactose uptake; that the glycolysis and fermentative pathways would be promoted and generated energy to support plantaricin production. The increase in glutamate metabolism and decrease in arginine biosynthesis may replenish the glutamate and then enhance the plantaricin yield, and the upregulation of the *agrA* and *oppA* genes may help transport PlnA and regulate plantaricin production. Our study provides a novel insight into the mechanisms of bacteriocin-producing bacteria's response to bacteriocin-inducing yeast and offers a reference for the interaction between *L. paraplantarum* and *W. anomalus*. In the future, a detailed study of the bacteriocin-inducing regulatory mechanism in co-culture could be undertaken using metabolomics methods.

## Data availability statement

The datasets presented in this study can be found in online repositories. The names of the repository/repositories and accession number(s) can be found in the article/Supplementary material.

## Author contributions

RN and GL conceptualized the idea and designed the experiments. RN wrote the manuscript. GL, RN, and YQ revised, make corrections, and approved the research article for publication. RN, ZZ, ZW, and HS performed the experiments. RN and ZZ analyzed the results. All authors contributed to the article and approved the submitted version.
