# Supplementary material for: Bacteriocin production enhancing mechanism of Lactiplantibacillus paraplantarum RX-8 response to Wickerhamomyces anomalus Y-5 by transcriptomic and proteomic analyses
Source: Front Microbiol. 2023 Feb 24;14:1111516. doi: 10.3389/fmicb.2023.1111516 (PMC9998909; doi:10.3389/fmicb.2023.1111516)
Supplement: Supplementary file 1 [file Data_Sheet_1.docx]

Supplementary Material

Inducing Bacteriocin Producing Mechanism of *Lactiplantibacillus paraplantarum* RX-8 Under Co-culture by Transcriptomics and Proteomics Analysis

Rong Nie ^†^, Zekang Zhu^†^, Yanwei Qi, Zhao Wang, Haoxuan Sun, Guorong Liu^*^

**^†^ These authors contributed equally to this work and share first authorship**

*** Correspondence:** Guorong Liu^*^: liuguorong1983@126.com

# Supplementary Figures


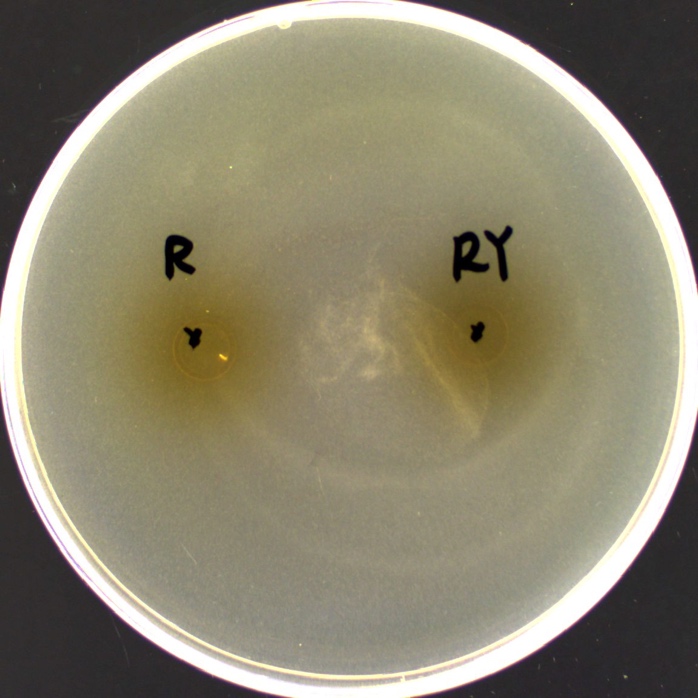


**Supplementary Figure 1.** **The inhibition zone of CFSs from mono-culture and co-culture against** ***Wickerhamomyces anomalus* Y-5.** R means mono-culture of *L. paraplantarum* RX-8 at 37°C for 24 h, RY means bacteriocin-inducing co-culture of *L. paraplantarum* RX-8 and *W. anomalus* Y-5 at 37°C for 24 h.


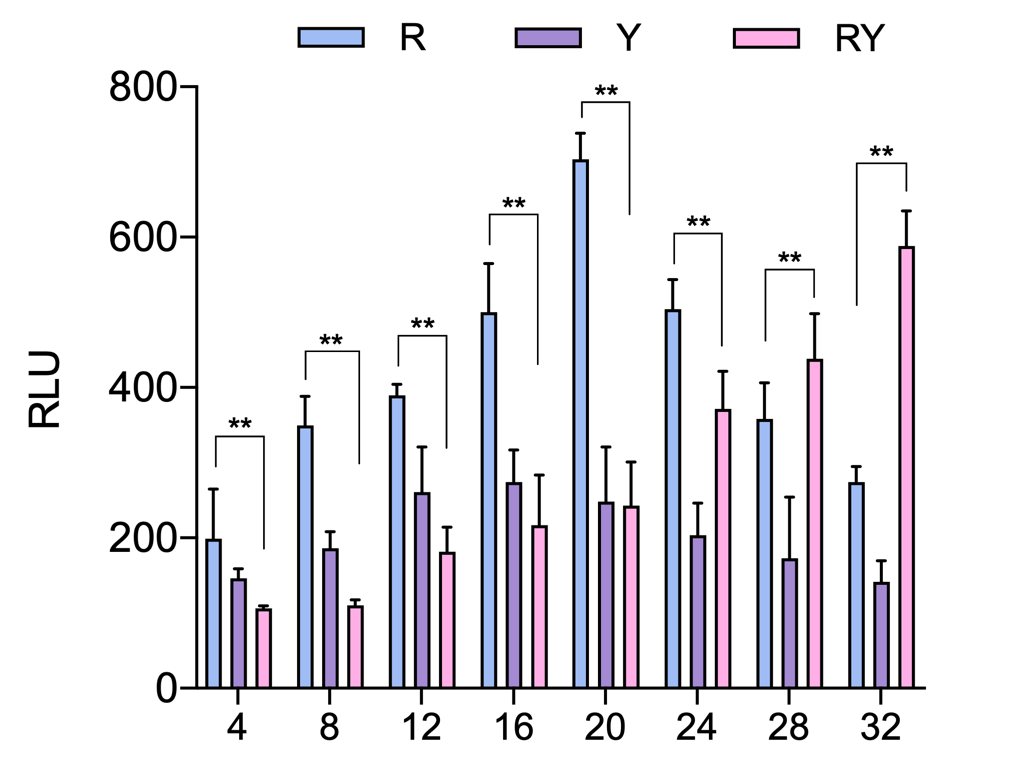


**Supplementary Figure 2.** **The change of AI-2 activity in mono-culture and co-culture.** R means mono-culture of *L. paraplantarum* RX-8 at 37°C in 4~32 h, Y means mono-culture of *W. anomalus* Y-5 at 37°C in 4~32 h, RY means bacteriocin-inducing co-culture of *L. paraplantarum* RX-8 and *W. anomalus* Y-5 at 37°C in 4~32 h. ** *P* < 0.01
